# A Methodological Framework for Aggregating Branded Food Composition Data in mHealth Nutrition Databases: A Case Presentation

**DOI:** 10.3390/nu18020359

**Published:** 2026-01-22

**Authors:** Antonis Vlassopoulos, Stefania Xanthopoulou, Sofia Eleftheriou, Ioannis Koutsias, Maria C. Giannakourou, Anastasia Kanellou, Maria Kapsokefalou

**Affiliations:** 1 Department of Food Science & Human Nutrition, Agricultural University of Athens, Iera Odos 75, 11855 Athens, Greece; sxanthopoulou@aua.gr (S.X.); ioanniskoutsias@aua.gr (I.K.); 2 Department of Food Science and Technology, Faculty of Food Science, University of West Attica, 12243 Athens, Greece; seleftheriou@uniwa.gr (S.E.); akanellou@uniwa.gr (A.K.); 3 Laboratory of Food Chemistry and Technology, School of Chemical Engineering, National Technical University of Athens, Iroon Polytechniou 5, 15780 Athens, Greece; mgian@chemeng.ntua.gr

**Keywords:** branded food data, mHealth, digital applications, generic food data, nutritional composition, data aggregation

## Abstract

**Background/Objectives**: Up-to-date, relevant and detailed food composition databases (FCDs) are a central component of mHealth apps. Thus, the expansion and/or update of such FCDs though the aggregation of branded food data (BFCDs) could prove as a cost-efficient methodology. However, a framework for data aggregation from BFCDs has yet to be documented. **Methods**: Products (*n* = 3988) available in the HelTH BFCD were grouped following a three-step process. Firstly, foods were grouped based on their name, and then the aggregated nutritional composition was tested for heterogeneity using a coefficient of variation cut-off of 20% followed by a search of the ingredient list and other product characteristics to identify descriptors that reduced heterogeneity. **Results**: Following a three-step process, *n* = 347 new generic food names were proposed, each derived from at least three branded products, of which *n* = 235 were populated with aggregated nutritional content values. We found that 95.3%, 88.6%, 86% and 82.6% of aggregated energy, protein, carbohydrate and sodium values, respectively, had a coefficient of variation <40%. Aggregated saturated fatty acid and total sugar values were less likely to fall in the homogeneity level (76.3% and 65.3%, respectively). The heterogeneity was concentrated in specific subcategories like baked goods, milk products and milk imitation products, primarily. **Conclusions**: BFCDs can be used as a resource to expand existing databases with relatively homogeneous and up-to-date nutritional composition data. The application of this framework on larger datasets could improve the generic food name yield and homogeneity and support mHealth apps and other uses.

## 1. Introduction

Digital applications to track and modify health indices and behaviors, including both electronic services (eHealth) and mobile apps (mHealth), are used by millions on a daily basis [1]. Evidence on the efficacy and usability of such apps is mounting and so is their prevalence in app stores (>60,000 versions) [2], targeting diverse population groups [3,4,5,6].

Despite their popularity and positive outcomes in certain domains of nutrition interventions, such as promoting behavioral change, mHealth applications for dietary assessment (e.g., diet tracking) continue to face critical validity and implementation challenges [7,8,9,10,11]. The nature of traditional food composition datasets (FCDs) and their distance from modern citizen science and big data methodologies have been identified as key hurdles in supporting digital nutrition solutions [12,13,14,15,16]. Research has showed that FCDs that (a) include branded food data, (b) allow for data entry through barcode scanning and, of course, (c) allow for easy mining from large datasets (10–120 thousand foods) are much more suitable for digital applications than traditional FCDs [11,17,18,19,20].

In this context, branded food composition databases (BFCDs), which track and compile the composition of individual foods as sold in the supermarket, are potentially powerful additions to mHealth apps [21,22,23,24]. Although currently limited in their prevalence, BFCDs are growing globally and are a key driver behind the proliferation of data points included in national food composition resources [21,22,23,24]. BFCDs allow for high volume data input [18,25,26,27], often resolving the need for manual data entry from users [11,17,18] or data borrowing from datasets of other countries [18]. Nonetheless, BFCDs are still required to work alongside and contribute towards large nationally representative FCDs [28,29].

A key limitation of BFCDs is the fact that despite the high granularity in food descriptions (e.g., oat-based cereal clusters with chocolate chips, sourdough wheat bread loaf sliced, cow milk strained yogurt 2% fat with strawberry jam), to date there has been a lack of a unified protocol for data aggregation. Unlike granularity, data aggregation is important when data points need to be expressed as average values of similar foods, called generic foods. Generic foods are the backbone of nutritional epidemiology, nutritional assessments and the national FCDs themselves [30,31]. Through data aggregation from BFCDs, one can expand national FCDs especially in contexts of limited resources [32,33,34], but most importantly in the case of mHealth apps, it allows for better data mining and better nutritional assessments and guidance. The development of systematic data aggregation principles is also needed in any artificial intelligence, machine or deep learning application linked to the prediction of the nutritional composition of foods, the proposal of food substitutions or digital assistance in nutritional assessments [35,36,37].

Although data aggregation might seem straightforward as a process, previous analyses of food products in the same category, e.g., in the breakfast cereal category, suggest that there are similarities as well as differences among products in this category regarding their total fat, saturated fat, sugar or salt content [33,34]. This suggests opportunities in aggregation but also the need for the development of a framework and the criteria for generating potential generic food descriptors. To date, to our best knowledge, there has been no documented methodology for data aggregation from BFCDs, and little is known about branded data aggregation’s ability to provide granular and homogeneous generic data. In the current study we will present the case of developing such a framework for data aggregation in the context of an mHealth application.

In brief, MedDietAgent, a context-aware mobile computing system aiming to promote adherence to the Mediterranean Diet and the Greek culinary culture, is a system that allows for personalized dynamic recommendations based on nutritional assessments and personal goals [38]. For MedDietAgent, there is a need to develop a tailored food composition dataset which would combine existing aggregate-level data for traditional foods and recipes [39] with the newly developed BFCD for Greece [40]. This approach should allow for both the mining of food-level compositions, where available, and also the creation of a more detailed list of aggregate food composition values to expand the current limited food list available on national FCDs.

The aim of the current study was to employ a mixed methodology, combining qualitative food name analyses, ontologies and statistical reconfigurations to test the capacity to (i) generate aggregated values from branded foods and (ii) ensure sufficient homogeneity to be used as generic food items.

## 2. Materials and Methods

### 2.1. Data Source

The first food composition database for branded packaged foods sold in Greece (HelTH) was used [40,41]. The HelTH is a dynamic food composition database currently in its third version, which collects and curates nutritional compositions and other data as provided on the package of branded foods sold in Greece. The version of the HelTH used in the current study included information for the nutritional composition, on-pack claims and quality indicators for 4851 products collected from November 2019 to October 2022. The products were categorized in food categories and food subcategories based on the European Food Information Resource’s (EuroFIR) classification system as indicated in the LanguaL^TM^ Food Description Thesaurus. The BFCD consists of five different files: description, nutrients, claims, allergens and a photobook. The “nutrients” file provides comprehensive data on the nutritional composition of foods as declared on packs. The HelTH curates the available data on the pack without an additional chemical analysis and without intervening with the declared values. Among the 4851 products available in the HelTH, *n* = 113 products were excluded due to a lack of images of all sides of the product’s package (*n* = 4738).

For the purpose of this analysis, a data quality check was introduced for improbable values by (i) the calculation of the energy content from the respective macronutrients with the carbohydrates and protein assigned to every 4 kcal per g, fat assigned to 9 kcal per g and fiber and polyols assigned to 2 and 2.4 kcal per g, respectively; (ii) the ratio of saturated fatty acids (SFAs) to total fat (TF); and (iii) the ratio of total sugars (TSs) to carbohydrates (CHO). Foods with a >20% difference between the declared on-pack and calculated energy or with SFA:TF or TS:CHO ratios larger than 1 and/or those that were considered as containing improbable values were excluded from the analysis. A total of *n* = 750 products were excluded from the analysis (*n* = 747 for energy, *n* = 2 for SFA:TF and *n* = 1 for TS:CHO). The final dataset included *n* = 3988 foods.

### 2.2. Methodology of Aggregated Value Derivation

**Figure 1:**
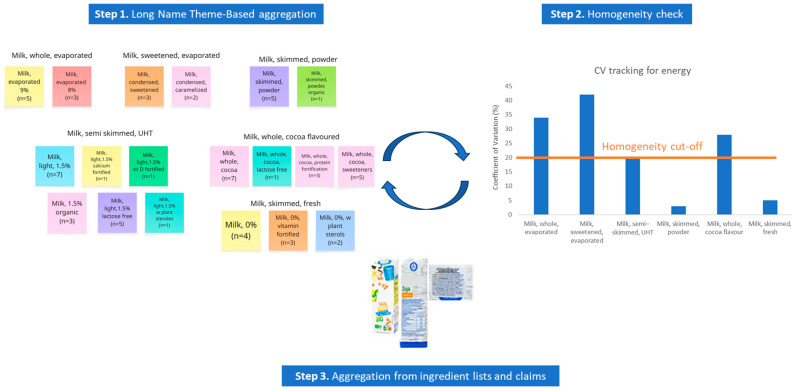
Three-step product aggregation process using the long name, statistically measured homogeneity (via the coefficient of variation) and elements found in ingredient lists and on-pack claims.

The aggregation of branded food data for higher level generic food descriptors was organized according to a mixed methods approach that applied a three-step iterative framework (Figure 1).

Firstly, the food description file was used, and more specifically the foods’ long name and food description per LanguaL^TM^ in food categories, subcategories, food groups and subgroups were used as detailed previously [40]. All foods were described and categorized in the HelTH following the LanguaL^TM^ ontology into 15 categories, 41 subcategories, 130 groups and 205 subgroups. At this first step, food names within each subgroup were screened to identify shared qualitative attributes like the animal or plant of origin, processing type, addition of flavors, etc. (Table 1). All food names were screened by two independent researchers using the Miro web-based software for the real-time grouping of foods. Researchers performed the groupings independently, and Miro workbooks were shared among them to identify areas of classification conflicts. Conflicts were resolved at the end of all classifications per food group. Conflicts were discussed in a group setting in the presence of a third researcher, during which each researcher presented arguments for their classification and the third researcher voted in favor of one of the two approaches (tie-breaker). In the case that the conflict impacted other groupings within the category, the classification process was updated and repeated.

Following the qualitative grouping based on the information available for the product’s long name, descriptive statistics were used to explore the homogeneity of the groups created in terms of their macronutrient content. For each group, the mean, standard deviation (SD) and coefficient of variation (CV = SD/Mean) were calculated for energy and macronutrients (total fat, saturated fatty acids, carbohydrates, total sugars, protein and sodium). A CV > 20% in at least one macronutrient or energy was considered as evidence of heterogeneity based on an arbitrary assumption inspired by the principles of identifying high inter-assay heterogeneity in analytical settings [42]. Heterogeneous groups were further examined in the third step, with the aim of identifying additional descriptors in the ingredient list or other elements of the packaging (e.g., content claims) that would explain the heterogeneity (Table 1). Once the new descriptors were identified, the initial groups were split further, and heterogeneity was tested once again. When high CVs persisted but the description and ingredients of the foods did not propose the need for further grouping, the heterogeneity was considered inherent, and the process of the descriptor identification was determined.

### 2.3. Statistical Analysis

Descriptive statistics were employed in this study. The mean, standard deviation (SD) and coefficient of variation (%) were calculated to describe the nutritional composition (per 100 g/mL), including the energy (kcal) and protein, total fat, saturated fat, carbohydrates, total sugars, fibers (g) and sodium (mg) for each new aggregated category. Statistical analyses were performed using IBM SPSS Statistics^®^ (version 27, Northridge, CA, USA). For categorical data, food categories, subcategories, groups, frequencies and relative frequencies were calculated.

## 3. Results

### 3.1. Data Aggregation to Derive Generic Food Names from Branded Data

Following data aggregation protocol, the *n* = 3406 foods with suitable long names for inclusion in the analysis could be aggregated into *n* = 1112 generic food names (Table 2). Foods with long names that suggested products that were multipacks of different food products (ham and cheese combos, crackers and jam combos, etc.) were excluded from the aggregation (*n* = 1332). The majority (22.9%) of generic food names were identified in the grain and grain product category, followed by milk, milk products or substitutes (14.1%); miscellaneous food products (13.8%); and sugar or sugar products (13.3%). At this point it is important to clarify that the food category of miscellaneous food products includes savory snacks, condiments, etc.

**Figure 2:**
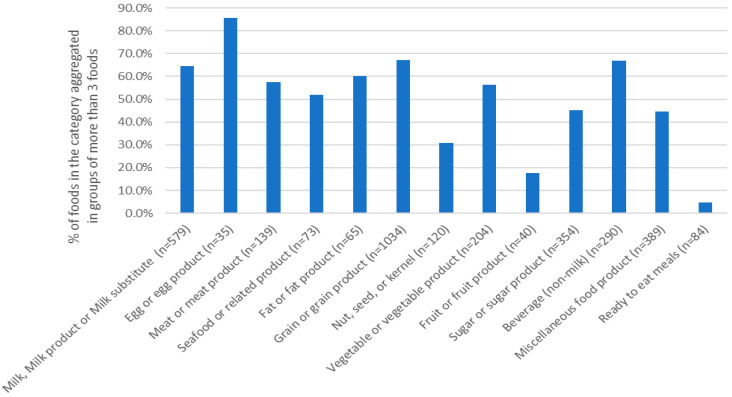
Percentage of products per food category that were aggregated under the same generic name in groups of ≥3 products.

In terms of the aggregation capacity per food category, eggs and egg products were reduced the most by 86% (from 35 products to five generic names), with 85.7% of all products in the category grouped under generic names with ≥3 products in them. Eggs in particular are a very homogeneous group in our database, as the key differences in egg products are the size and the manufacturer, with limited permutations for non-hen eggs and egg whites sold as a liquid product. This intrinsic characteristic of this category does not contradict the finding; it rather highlights the ease of aggregation in non-processed food categories. On the contrary, ready-to-eat meals were the least reduced category (14% reduction), with 76% of all products creating one generic name and only 4.8% of products being able to be aggregated in groups of ≥3 products (Figure 2). Fruit and fruit products, as well as nuts, seeds and kernels, showed the next highest aggregation difficulty, with 17.4% and 30.8% of products being able to be aggregated in groups of ≥3 products. For the majority of the categories, 44.5% to 67.2% of the products could be aggregated in groups of ≥3 products, suggesting acceptable aggregation potential. In total, 72.4% of all products in the HelTH database could be aggregated under generic names that would represent at least three products. The reduced aggregation capacity for ready-to-eat meals, fruits and nuts was primarily a function of the extreme diversity between the foods available in the database. Especially for ready-to-eat meals, 72 out of the 84 foods available represented a unique recipe or food offering, indicating that the specific category would require much larger sample sizes to be effectively aggregated. The same can be said for fruits and, to a lesser extend, for nuts, where again the relatively small sample size and the variability of the foods included in the categories did not facilitate aggregation.

### 3.2. Compositional Homogeneity

In the analyses for the homogeneity of the compositional value of the aggregated food products, only entries with the relevant macronutrient data were included. That included energy *n* = 986, protein *n* = 974, total fat *n* = 988, SFA *n* = 960, CHO *n* = 966, TS *n* = 960 and sodium *n* = 948, while complete data for all the above nutrients were available for *n* = 910 products. Only the *n* = 235 generic food names populated by ≥3 products with nutritional composition values for all nutrients of interest were analyzed for homogeneity.

**Figure 3:**
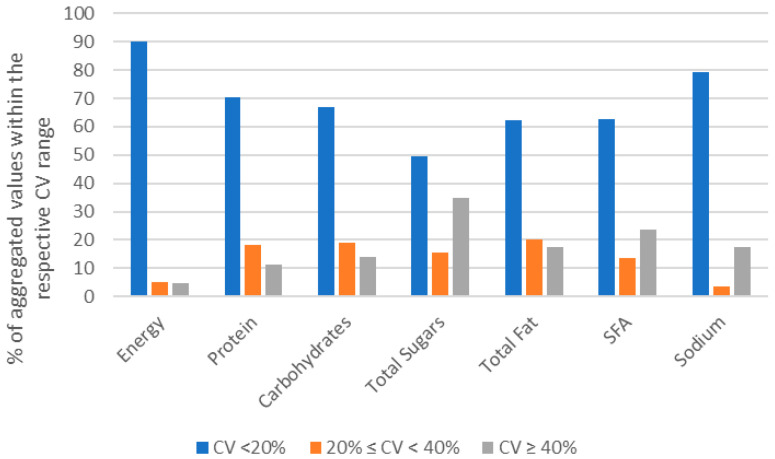
Percentage of aggregated values calculated for energy, protein, carbohydrates, total sugars, total fat, saturated fatty acids (SFAs) and sodium per coefficient of variation (CV) grade.

When generating nutritional composition values from aggregated branded food data, for each generic food proposed, 90.2% of energy content values and 79.2% of sodium content values showed a coefficient of variation (CV) < 20%, indicative of high homogeneity in the current analysis (Figure 3). The total fat, SFAs and carbohydrates showed a similar capacity to generate homogeneous content values for aggregated foods, with 62.3, 62.7 and 66.9% of generic foods with a CV < 20% for these nutrients, respectively. The aggregated protein content values were considered highly homogeneous for 70.3% of generic foods. On the contrary, total sugar values were more prone to increased heterogeneity, with 49.6% of aggregated content values with a CV < 20% and 34.7% with a CV > 40% (Figure 3).

**Figure 4:**
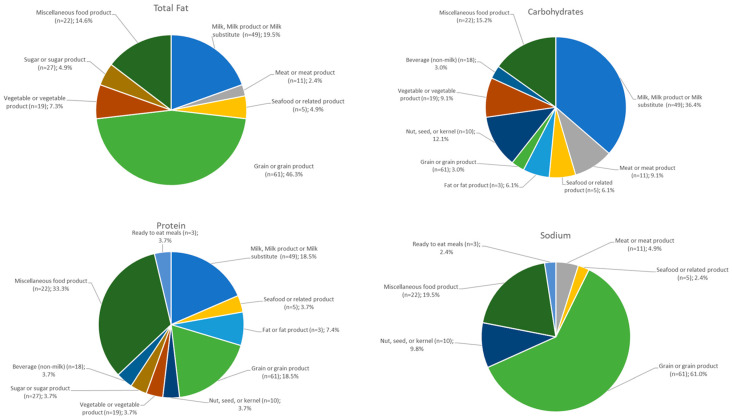
Distribution of aggregated content values with CV > 40% for protein, carbohydrates, total fat and sodium among the food categories.

Aggregated values with high heterogeneity (CV > 40%) tended to cluster in specific food categories like grains and grain products; milk, milk products and substitutes; miscellaneous foods; and non-milk beverages (Figure 4).

There were clear patterns of high heterogeneity in specific nutrients highlighted per food category (Figure 4), with the total fat being heterogeneous primarily in grains and grain products, a category that includes fine bakery ware. Similarly, carbohydrates were heterogeneous in milk, milk products and substitutes, while the protein content was heterogeneous in miscellaneous food products, an intrinsic characteristic of a very diverse category. At the same time, protein heterogeneity was seen in milk, milk products and substitutes, primarily due to milk substitutes having different protein contents. However, when the absolute protein content differences were studied within even the high heterogeneous groups those were less than 1 g per 100 g. The same can be said for grain and grain products where the protein content is generally low and small differences can translate to high CVs. Differences in sodium were clustered in food categories with a high presence of mixed dishes or complex foods like meat products, grains (baked goods), ready-to-eat meals and miscellaneous foods (dressings, sauces, bouillons). The limited representation of ready-to-eat meals in the highly heterogenous food groups can also be explained by the low aggregation capacity mentioned earlier for this food category.

The derivation of aggregated nutritional composition values for generic foods populated by *n* = 2 was not carried forward as 49.5% to 83.5% of nutrient values derived would have a CV ≥ 40%.

## 4. Discussion

This is to our knowledge the first attempt to propose and apply a data aggregation process from branded food composition data towards generic food items. In this study, using the 2022 version of the HelTH BFCD [40,41], the authors managed to identify *n* = 1112 potential generic food descriptors, of which 31.2% were derived by groups of at least three similar branded foods. The processes showed that the modern foodscape includes a large variety of foods, and traditional generic food descriptors may be limited or describe food choices using descriptors that are too broad. In fact, in countries like Canada, the Netherlands, the United Kingdom, the United States of America and Slovenia, modern food composition databases show a proliferation of food products available in the market, which can even surpass 100,000 foods [22,23,24,30,31]. In our case, despite lower data volumes, the number of potential generic food names (*n* = 1112) exceeded existing infrastructures [39] and even introduced new characteristics by which foods should be described. Descriptors like with non-caloric sweeteners, with added protein, multiple flavors, with bioactive ingredients and different levels of fat content and a vast range of multi-ingredient foods were among the long list of elements to be considered when grouping foods together.

Despite this large variety and the relatively small sample size of the HelTH compared to other BFCDs, sufficient composition data were available for 235 generic food descriptors to derive aggregated content values for energy, total fat and saturated fatty acids, carbohydrates and total sugars, protein and sodium. In the context of mHealth apps, the nutrients available cover the basic user needs [9,10,25,27,28,29,43]. As previously mentioned, the derivation of aggregated values for other nutrients like fiber, polyols and micronutrients would have been more challenging due to data completeness issues linked to the nature of BFCDs [40,44]. In the current study, data completeness constraints led to the exclusion of 21% of all potential generic food names from ≥3 products at the nutritional composition homogeneity stage. Nonetheless, this was not associated with the capacity to derive homogeneous aggregated nutritional composition values for the remaining descriptors.

Overall, aggregated energy values showed significant homogeneity for a little more than 90% of all proposed generic food terms. Sodium and protein contents were the next most likely nutrients to provide data with homogeneous aggregated contents, followed by carbohydrates. On the contrary, total sugar and SFA contents were much more likely to be heterogeneous, reflecting the wide variation in ingredients and formulation practices used across recipes, such as differences in the amount and types of added sugars (e.g., reduced-sugar products, use of non-nutritive sweeteners) and types of fats (e.g., butter, cream, vegetable oils). From the proposed generic food names, 34.7% and 23.7%, respectively, showed heterogeneous (CV > 40%) total sugar and SFA contents. This was particularly true for fine bakery ware, dairy products (especially flavored yogurts, milk drinks, dairy desserts) and dairy imitations. This heterogeneity is most likely intrinsic to these categories, as shown from market-wide surveys in the UK, Canada, Sweden and our previous analyses [41,45,46,47,48], and is not a caveat of the aggregation framework. Similarly, in the category of miscellaneous food products, large CVs were documented, but this category is inherently highly heterogeneous, ranging from bouillon cubes to crisps and other savory snacks.

The heterogeneity seen in the miscellaneous food group can in fact be attributed to the LanguaL ontology used in the current study. LanguaL is an ontology based on the technological and intrinsic characteristics of foods rather than their role in the diet, and hence it sometimes groups together foods with very different nutritional compositions. As the ontology is a central pillar in the aggregation framework, future work should investigate whether different ontologies like FoodEx2, which allows for the use of more varied facets, could allow for a better organization of the products along ontological branches. This improved organization might facilitate the process of deriving more homogeneous aggregated nutritional composition values and might even allow us to identify appropriate points at which aggregation should stop to avoid overly broad food terms.

For mHealth apps, the availability of well-defined generic food descriptors derived from BFCDs is particularly relevant, as users of dietary assessment applications often record foods without specifying brand names or encounter products that are not represented in branded databases [26]. The aggregation framework proposed here supports the translation of branded food information into generic food names that better reflect real-world user input while preserving nutritional data validity. This is especially important for long-term self-monitoring and population-level analyses, where consistency over time is required despite rapid changes in the food supply [22,31]. By enabling the use of nutritionally homogeneous generic food items, this approach may reduce user burdens, limit misclassification and improve the comparability of dietary data collected through mHealth tools [29]. Data aggregation is proposed to work alongside each food’s unique characteristics (claims, brand name, etc.), as the access to foods on a brand level is especially important when users need to identify culturally and religiously relevant options within the same food group (e.g., halal, kosher)—descriptors that might not impact the nutritional composition but are central to the relevance of the app for the user.

The framework presented herein is most likely one approach among many used by data specialists; however, the lack of peer-reviewed, open access documentation of the data aggregation methodologies used is hampering knowledge sharing and the establishment of a unified methodology. More and more national agencies are relying on BFCDs for their food epidemiology activities [24,49], and as data aggregation is a common step performed in any analysis or exploitation of BFCDs, it is important to promote the sharing of those methodologies. This study presents a step in that direction. The framework presented in the current study is tailored to the data available in the HelTH 2022 version, and the descriptors generated are data-driven using the overarching three-step methodology, which can be applied in any dataset.

Of course, the results are not without limitations. The large number of generic food descriptors that remain unpopulated in terms of nutritional content values are primarily due to the small sample size of the BFCD used. Future iterations of the framework should aim to be repeated in larger datasets, like the USDA branded food product database [24] or the OpenFood Facts database [50], in the hope of increasing the number of foods identified under each proposed generic food descriptor. To some extent, some elements of the nutritional composition heterogeneity among the aggregated content values could also be explained by the small sample size. Updated versions of the framework could incorporate statistically driven descriptor generation (e.g., reduced sugar, sodium or fat variants) even in the absence of explicit on-pack claims. An important finding in that regard was the different ways in how foods sold in brine, syrup or oil report nutritional values, depending on whether the surrounding liquid is included. In our case, without a statistical analysis there were no indications on pack to explicitly state whether the consumer is expected to consume the product as sold or after removing the liquids. This highlights the importance of statistical validation in identifying hidden sources of variability. Despite limited nutritional content data access (only those mandated to be declared on pack), aggregated micronutrient and fiber values could be added to the framework if a separate consideration is made for fortified products to be described as a separate generic term. Of course, the consideration of a CV > 20% as evidence of heterogeneity should also be mentioned as a limitation, since this threshold is primarily inspired by the common practice in analytical settings [42], but its use in this context is arbitrary.

A key consideration linked to the use of BFCDs is the reliance of declared on-pack data, which is limited to a set number of macronutrients primarily, and data on micronutrients are sparse and linked to health and nutrition claims. In this context, analyses that utilize fiber, micronutrient, trans fat, added sugar, etc., are severely impacted by the limited data completeness [22,40,41]. Similarly, a common concern with labeled data is the inability to verify the values against a laboratory analysis. Although research in the field indicates a good agreement in spot sample analyses, there is always a risk of identifying a proportion of foods with erroneous values declared [51,52]. Although this does not impact the average values derived by the proposed aggregation framework, an extreme case analysis should be considered to understand the potential clustering of errors among specific food groups or retailer sizes.

Finally, even if users are skeptical of relying on branded data for the update of nutritional composition values for generic foods, the qualitative steps of the proposed framework could be used as a method to inform a sampling methodology for the creation of a chemical-analysis-based FCD. Food names, ingredient lists and on-pack claims can be used to create a list of the relevant descriptors one should consider when aiming to collect a representative sample of the modern foodscape. The current work showed that the nearly 4000 branded foods analyzed would require a total of 1112 different descriptor combinations to be grouped appropriately. These descriptors are food category- and food group-specific.

Future research in the field should include the validation of this framework in larger datasets, especially those populated with ready-to-eat meals, fruits and nuts. It may also include the use of machine learning- or AI-assisted methodologies to automate the thematic analysis and explore the potential to develop micronutrient prediction algorithms using the proposed ontology and the ingredient list data.

## 5. Conclusions

With the aim of developing a nutritional composition database for an mHealth application, we aggregated branded food composition data from the branded food database HelTH. In the case of the HelTH, the process can produce >300 food codes with values from more than three products. These generic foods represent >70% of all foods in the HelTH database. For the majority of those foods (>80%), the derived energy, protein, carbohydrate and sodium content values would have acceptable homogeneity. The derivation of aggregated content values for total sugars and SFAs is more prone to higher heterogeneity. Herein, we present the methodological framework for food composition value aggregation and demonstrate that the utilization of branded food composition databases to generate aggregate nutritional composition values for generic foods is a viable methodological process.

## Figures and Tables

**Table 1:** Indicative examples of the aggregation descriptors expected to be employed at each level of qualitative grouping and the respective generic food names to be generated.

Food Categories	Indicative Aggregation Descriptors Identified in the Food’s Long Name	Indicative Aggregation Descriptors Identified from Ingredient Lists or Other Characteristics	Example Generic Food Names
Milk, milk product or milk substitute	Animal of origin (cow, sheep, goat, donkey, mixed) Processing (pasteurized, UHT, condensed, powder, fermented) Fat content (skimmed, semi-skimmed, full fat or numerical values) Flavored (plain, chocolate, other flavors)	Sweeteners (sugar, non-nutritive sweeteners, honey, juices, jams, purees) Protein fortification (whey, soya, pea)	Yogurt, cow milk, strained, 0% fat, plain Milk, cow, pasteurized, 2% fat, chocolate, with stevia Almond drink, low fat, UHT, chocolate, pea protein fortified, with sweeteners
Fat or fat product	Animal or plant (or mixed) Solid, liquid, spreadable	Bioactives (stanols, sterols) Secondary ingredients (yogurt)	Butter, cow, spreadable, unsalted, with yogurt Margarine, sunflower and olive oil mix, spreadable, unsalted, with plant sterols
Grain or grain product	Main grain (wheat, oat, spelt, multigrain) Secondary ingredients (nuts, fruits, seeds) Processing (baked, dried, wholegrain) Coatings or fillings	Sweeteners (sugar, non-nutritive sweeteners, honey, juices, jams, purees) Fortification (protein, fiber)	Bread, multigrain, wholegrain, sliced Bread, wheat, white, with glucomannan, sliced Biscuits, wheat, plain, chocolate coating
Vegetable or vegetable product	Processing (canned, frozen, dried) Liquid matrix (brine, water, syrup, oil) Secondary ingredients (spices, fillings)	Recommended consumption (with or without liquid matrix)	Spinach, leaves, frozen Mushrooms, shitake, dried, with spices Asparagus, white, in brine, strained Peppers, red, roasted, with cream cheese stuffing, in oil, non-strained

**Table 2:** Number of generic names identified and prevalence of generic names that were populated by 1, 2 or ≥3 products per food category.

Food Categories	N Generic Names	N Generic Names from 1 Product	N Generic Names from 2 Products	N Generic Names from ≥3 Products
Milk, milk product or milk substitute (*n* = 579)	157	69	24	64
Egg or egg product (*n* = 35)	5	2	0	3
Meat or meat product (*n* = 139)	45	24	7	14
Seafood or related product (*n* = 73)	27	15	4	8
Fat or fat product (*n* = 65)	22	12	2	8
Grain or grain product (*n* = 1034)	255	113	42	100
Nut, seed or kernel (*n* = 120)	57	31	13	13
Vegetable or vegetable product (*n* = 204)	67	31	11	25
Fruit or fruit product (*n* = 40)	25	17	4	4
Sugar or sugar product (*n* = 354)	148	91	23	34
Beverage (non-milk) (*n* = 290)	78	33	9	36
Miscellaneous food product (*n* = 389)	154	89	31	34
Ready-to-eat meals (*n* = 84)	72	64	4	4
Total *n* records	1112	591	174	347

## Data Availability

The original contributions presented in this study are included in the article. Further inquiries can be directed to the corresponding authors.

## Abbreviations

The following abbreviations are used in this manuscript:

BFCD	Branded Food Composition Database
FCD	Food Composition Database
EuroFIR	European Food Information Resource
SFA	Saturated Fatty Acid
TF	Total Fat
TS	Total Sugar
CHO	Carbohydrate
